# Comparative Lipidomic Analyses Reveal Different Protections in Preterm and Term Breast Milk for Infants

**DOI:** 10.3389/fped.2020.00590

**Published:** 2020-10-20

**Authors:** Liping Xu, Wenjuan Chen, Xingyun Wang, Zhangbin Yu, Shuping Han

**Affiliations:** ^1^Department of Pediatrics, Zhangzhou Affiliated Hospital of Fujian Medical University, Zhangzhou, China; ^2^Department of Pediatrics, Women's Hospital of Nanjing Medical University, Nanjing Maternity and Child Health Care Hospital, Nanjing, China

**Keywords:** colostrum, lipids, LC-MS/MS, neonate, preterm

## Abstract

**Aim:** Neonates are notably vulnerable, however they have improved outcomes if they are fed human milk. Human milk lipids constitute the primary constituents of human milk and serve a pivotal role in safeguarding infants from diseases. We assessed the lipid differences between preterm and term human milk and predicted the prospective impacts of these lipids on the development of neonates.

**Methods and results:** We collected colostrum from healthy breast-feeding mothers who had delivered either term or preterm infants. We analyzed the lipid profiles of preterm, as well as term human milk using an LC-MS/MS metabolomics strategy. The orthogonal partial least-squares discriminant analysis score plots revealed remarkable distinction of lipids in preterm and term human milk. In total, 16 subclasses of 235 differential lipids (variable importance in projection > 1, *P* < 0.05) were identified. Notably, phosphatidylethanolamine and phosphatidylcholine were robustly increased in preterm human milk, while diacylglycerol and ceramide were markedly decreased in preterm human milk. Pathway analysis revealed that these dysregulated lipids are closely associated with glycerophospholipid metabolism, sphingolipid metabolism, Reelin signaling in neurons, and LXR/RXR activation.

**Conclusion:** The results show that the lipids in preterm and term human colostrum vary, which may be critical for neonatal development.

## Introduction

Human milk constitutes an optimal supply of nutrition and bioactive components for infants (1). Furthermore, human milk feeding is known to have several important specific protective actions and is recommended for preterm infants (2). Preterm neonates that consume human milk have improved immunity (3), decreased morbidity and mortality associated with necrotizing enterocolitis (4), better neurodevelopmental outcomes, and improved long-term health outcomes (5, 6). However, human milk composition is variable during the 1st weeks of life because the nutritional needs of preterm infants are different from those of term infants (7). Previous studies have investigated the constitution of preterm, as well as term human milk, indicating that significant differences are important for infant development (8).

Lipids constitute the majority of the macronutrient components in human milk and exert a nutritional effect. In addition, human milk lipids also play specialized roles in gastrointestinal function, infant growth, neurodevelopment, and immunity (9). Lipidomics is a powerful analytical approach and can be used to inspect the lipid metabolic reaction of living systems in intricate biological specimens (10). Recently, lipidomics has made significant research advancements on human milk, due to its great advances of chromatography, as well as mass spectrometry approaches (11). Among these analytical approaches, LC-MS/MS is extensively applied in lipidomics, particularly for identifying untargeted lipid profiles, due to its high resolution and preciseness (12). A previous LC-MS/MS study reported numerous differential medium-chain lipid species, including phosphatidylethanolamine (PE), phosphatidylcholine, diacylglycerol (DG), and prostaglandin (PG), between human milk and bovine or caprine milk (13). However, few studies have investigated the lipid profiles of preterm and term human milk using LC-MS/MS. Therefore, the identification and clarification of lipid expression profiles in preterm and term human milk are beneficial for understanding the protective impact of human milk on infants.

In this study, we aimed to assess differences in the lipidomic profiles of preterm, as well as term milk and to estimate the promising effects of these differential lipids on the development of neonates.

## Methods

### Sample Collection and Ethics Statement

We collected colostrum samples from healthy lactating mothers who had given birth to either term (37–41 weeks), or preterm infants (32–36 weeks) at the Women's Hospital of Nanjing Medical University. Informed consent was submitted by the mothers of all the infants. The Human Research Ethics Committee of Women's Hospital of Nanjing Medical University ratified this study (Permission Number [2015] 88). Six of the mothers were assigned to the preterm group, and the other six mothers were assigned to the term group.

### Metabolite Extraction

We transferred 100 microliters of each sample to a tube, and added 480 μL of extract solution (MTBE: methanol = 5: 1). Subsequently, we sonicated the samples for 10 min, then followed this with 1 h incubation at −40°C, and then 15 min centrifugation at 3,000 rpm, 4°C. A total of 350 μL of supernatant was further transferred to a fresh tube, followed by drying at 37°C in a vacuum concentrator. We reconstituted the dried samples in 100 μL of 50% methanol in dichloromethane. Then, we centrifuged the constitution at 12,000 rpm for 15 min at 4°C, and then transferred 75 μL of the supernatant into a glass vial for LC-MS/MS analysis. We prepared the quality control sample through blending equal aliquots of the supernatants of all the samples.

### LC-MS/MS Evaluation

We accomplished UHPLC dissolution on a 1,290 Infinity series UHPLC system (Agilent Technologies), fitted with a Kinetex C18 column (2.1 ^*^ 100 mm, 1.7 μm, Phenomen). The mobile phase A constituted water (40%), acetonitrile (60%), and ammonium formate (10 mmol/L) while the mobile phase B comprised of acetonitrile (10%) and isopropanol (90%), which included 50 mL of ammonium formate (10 mmol/L) for every 1,000 mL of the mixed solvent. We conducted the analysis by employing the following elution gradient: 0–12.0 min, 40–100% B; 12.0–13.5 min, 100% B; 13.5–13.7 min, 100–40% B; and 13.7–18.0 min, 40% B. The column temperature was 45°C. We set the autosampler temperature and the injection volume at 4°C, and 2 μL (pos) or 2 μL (neg), respectively.

We utilized a triple TOF mass spectrometer because of its capacity to realize MS/MS spectra on data-dependent rationale during an LC/MS assay. In this mode, the acquisition software (Analyst TF 1.7, AB Sciex) continually assessed the entire scan survey MS information as it acquired and stimulated the collection of MS/MS spectra subject to the preselected maxims. During every cycle, the most intense 12 precursor ions with intensities higher than 100 were selected for MS/MS at a collision energy of 45 eV (12 MS/MS events with accumulation time of 50 ms each). We set the ESI source parameters as: gas 1, 60 psi; gas 2, 60 psi; curtain gas, 30 psi; source temperature, 600°C; de-clustering potential, 100 V; and ion spray voltage floating, 5,000 or −4,500 V in positive or negative modes, respectively.

### Statistical Analysis

Using R, an in-house algorithm called LipidAnalyzer for automatic data analysis was developed (14). Employing the “msconvert” of the ProteoWizard (version 3.0.6150), we converted the raw data files into the mzXML format. After that, we fed the mzXML files into LipidAnalyzer for data analysis (15). Firstly, we employed the CentWave algorithm in XCMS to apply peak detection to the MS1 data. With the MS/MS array, lipid determination was attained via a spectral match by utilizing a local MS/MS spectral library. We applied the orthogonal partial least squares discriminant analysis (OPLS-DA) to the unit variance-scaled spectral data for visualizing the differences between preterm and term human milk. The coefficient loading plots and variable importance in projection (VIP) of the OPLS-DA model were employed in identifying the spectral variables accounting for sample dissolution. The VIP scores ranked the components according to their importance for the observed separation. Statistical distinctness was established using the Student's *t*-test, with *P* < 0.05 signifying significance (16). We conducted pathway assessments in MetaboAnalyst (http://www.metaboanalyst.ca/). Furthermore, ingenuity pathway analysis (IPA, http://www.qiagen.com/ingenuity/) was also applied to decipher the molecular interaction networks that are dysregulated in preterm and term human milk.

## Results

### Clinical Characteristics

The clinical features of the enrolled study participants are indicated in Table 1. The gestational ages of the term group and the preterm group were 39.16 ± 0.65 and 34.02 ± 0.71 weeks in the term group and the preterm group, respectively (*P* < 0.05). The birth weight of infants was remarkably lower in the preterm group (2360.00 ± 246.09 g) than in the term group (3240.00 ± 306.46 g, *P* < 0.05). The average birth length was 49.83 ± 0.41 cm in the term group and 46 ± 1.67 cm in the preterm group (*P* < 0.001). However, there were no remarkable differences in maternal age, body mass index, delivery type, or milk collection time between the term and preterm groups. Human milk samples were collected at 4.17 ± 1.72 d (term group) and 4.00 ± 1.67 d (preterm group, *P* > 0.05). The subjects were healthy mothers without maternal complications or bad habits. Among them, three mothers gave birth prematurely due to premature rupture of the membrane, while one mother had central placenta previa. The cause of premature delivery of the other two mothers is unclear.

**Table 1:** Maternal and infant characteristics.

**Characteristics**	**Term**	**Preterm**	***P*-value**
**Infants' Characteristics at Birth**			
Gestational age (weeks)	39.16 ± 0.65	34.02 ± 0.71	<0.001
Birth weight (g)	3240.00 ± 306.46	2360.00 ± 246.09	<0.001
Birth length (cm)	49.83 ± 0.41	46 ± 1.67	<0.001
**Maternal Characteristics**			
Maternal age (years)	28.83 ± 6.08	30.83 ± 4.71	0.538
BMI at birth (kg/m^2^)	26.47 ± 4.22	25.49 ± 3.28	0.663
GDM	0	0	
PIH	0	0	
Smoking history	0	0	
Alcoholism history	0	0	
Cesarean section (%)	33.33	50	>0.999
Milk collection time (postpartum days)	4.17 ± 1.72	4.00 ± 1.67	0.868

*Values were represented as mean ± SD (n = 6). Student t-test and chi-square test was used for statistical analysis*.

*BMI, body mass index; GDM, gestational diabetes mellitus; PIH, pregnancy-induced hypertension*.

### Multivariate Statistical Analysis of Lipids

**Figure 1:**
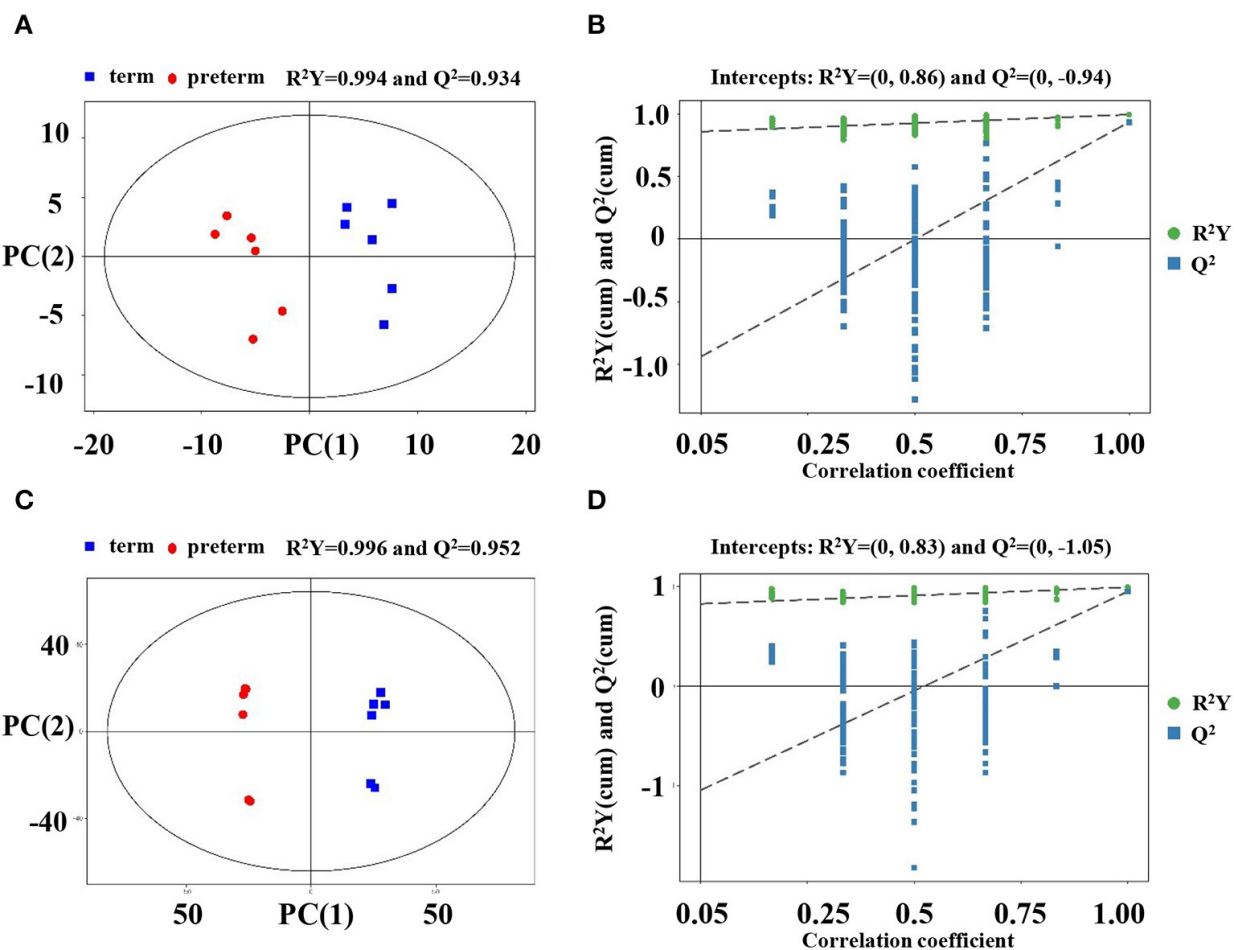
Score scatter plot and permutation test of the OPLS-DA model. **(A)** Score plots were generated from the LC-MS/MS data of preterm and term human milk lipidomic profiles in the positive ionization mode. The x axis and y axis represent the first and second principle components, respectively. The red and blue colors indicate respective groups. **(B)** Permutation tests were obtained from LC-MS/MS data in the positive ionization mode. The x axis and y axis of the permutation test represent the correlation coefficient and value of R^2^Y and Q^2^, respectively. **(C)** Score plots were obtained in the negative ionization mode. **(D)** Permutation tests were obtained in the negative ionization mode. OPLS-DA, orthogonal partial least-squares discriminant analysis.

Multivariate assessment, OPLS-DA, was employed to analyze the differences in preterm and term human milk lipids. OPLS-DA constitutes a supervised trend identification approach that allows for sample classification and removes unassociated noise data from the dataset. OPLS-DA was determined to additionally verify the dissolution of the metabolic profiles between the term and preterm human milk arms. The parameters R^2^Y, as well as Q^2^, were >0.5 in the positive and negative ionization modes, implying that the OPLS-DA model was well-determined. In the OPLS-DA score plots, the samples from the term and preterm human milk arms were completely separated (Figures 1A,C), further positing that there were distinct differences in the metabolic features of human milk. A permutation examination of the OPLS-DA model was proceeded to further verify the model. The R^2^Y and Q^2^ intercept values were (0, 0.86) and (0, −0.94), respectively, in the positive ionization mode (Figure 1B), and (0, 0.83) and (0, −1.05), respectively, in the negative ionization mode (Figure 1D). The results showed that R^2^Y and Q^2^ were the greatest in the current sample clustering, implying that our OPLS-DA model was robust, without overfitting. The low Q^2^ intercept values demonstrated the robustness of the model, and thus revealed authenticity, as well as a low risk of overfitting.

### Different Lipids Observed in Preterm and Term Human Milk

**Figure 2:**
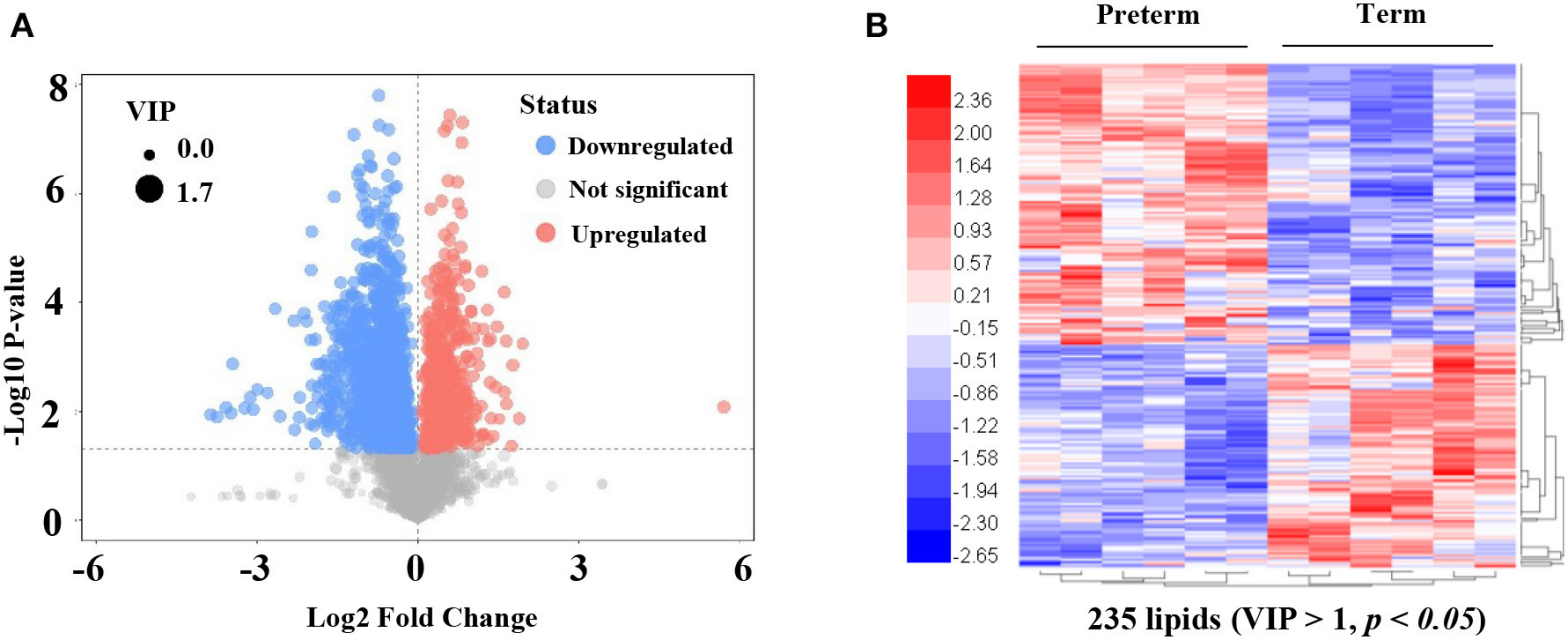
Significantly different lipids in preterm and term human milk. **(A)** The volcano plot is used for assessing significantly different lipids. Blue dots indicate downregulated lipids in the preterm group; red dots indicate significantly upregulated lipids in the preterm group; gray dots indicate the lipids that were not significantly changed in the preterm group. The size of dots indicates VIP value. **(B)** The heatmap of hierarchical clustering analysis is used for assessing significantly upregulated and downregulated lipids. Increased and decreased lipid levels are depicted by red and blue colors, respectively. The dendrogram was constructed based on the lipid intensity. VIP, variable importance in projection.

An LC-MS/MS strategy was used to determine the lipid profiles of preterm, as well as term human milk. Based on the results of the LC-MS/MS analysis, 4,686 lipids were found (Figure 2A). After eliminating the lipids identified in both the positive, as well as the negative ion mode repeatedly, 235 lipids (VIP > 1, *p* < 0.05) were remarkably different when comparing the preterm and term human milk groups, including 131 upregulated lipids and 104 downregulated lipids in the preterm group (Figure 2B). The top 30 markedly up modulated and down modulated lipids in the preterm arm can be found in Table 2. All of the markedly different lipids are shown in Supplementary Table 1.

**Table 2:** Top 30 significantly upregulated and downregulated lipids in preterm group.

**Name**	**Preterm**	**Term**	**VIP**	***P*-value**
**Upregulated**				
HexCer(d18:1/16:0)	8.67274E-05	5.72774E-05	1.736105792	3.6502E-08
CerP(d18:1/24:4)	4.04108E-05	2.26931E-05	1.711457827	4.96389E-08
PE(24:1/10:0)	0.000404915	0.000242691	1.715919004	6.17433E-07
PE(P-18:0/22:6)	9.90662E-05	7.00258E-05	1.680976896	5.75733E-06
PE(22:2/18:4)	0.000269758	0.000178651	1.679793437	7.29514E-06
PC(16:0/22:6)	0.000132881	7.61279E-05	1.696483018	9.66967E-06
PC(14:0/24:4)	0.000526544	0.000370156	1.648130565	1.30329E-05
PE(26:0/6:0)	2.81898E-05	2.13804E-05	1.63344555	2.37242E-05
PC(11:0/22:5)	4.236E-06	1.84932E-06	1.661793221	2.67364E-05
PE(22:5/16:1)	0.000129993	8.98846E-05	1.656433636	2.8123E-05
PC(14:1/24:4)	7.79234E-05	4.79878E-05	1.635165441	3.04858E-05
TG(18:1/18:1/18:3)	0.029991392	0.026391194	1.642302195	4.08878E-05
TG(16:1/16:1/20:4)	0.003456627	0.002466947	1.643141563	4.09498E-05
PI(14:1/22:2)	5.70714E-05	3.73758E-05	1.588697012	4.58621E-05
TG(18:2/18:2/18:2)	0.021745766	0.016665989	1.643068489	5.82604E-05
PC(17:0/26:0)	2.48972E-05	1.89917E-05	1.623936409	5.8378E-05
PC(22:5/16:1)	1.53022E-06	9.70095E-07	1.617607129	8.36243E-05
PC(18:2/22:6)	1.54571E-05	9.05764E-06	1.6402689	8.64854E-05
PI(8:0/26:2)	5.80489E-06	4.46149E-06	1.58495642	0.000108249
TG(16:1/18:2/18:2)	0.016478758	0.012703989	1.583927911	0.000110253
PE(8:0/26:2)	9.80808E-06	7.50189E-06	1.587356621	0.000130378
TG(18:2/18:2/18:3)	0.005508045	0.003539889	1.625897063	0.000139788
TG(16:1/18:1/18:2)	0.037039637	0.031851559	1.585365435	0.000155455
PE(24:0/10:0)	8.19788E-05	5.89761E-05	1.528237581	0.000176509
PC(18:3/22:6)	9.36971E-06	5.21967E-06	1.471141492	0.000218238
PC(26:2/6:0)	6.98558E-06	3.3344E-06	1.512640051	0.00029819
PE(P-16:0/22:6)	0.000201783	0.000146023	1.572414719	0.000324988
PC(17:0/26:1)	3.33652E-05	2.0138E-05	1.662093778	0.000448166
PE(22:4/18:3)	0.000299337	0.000227105	1.5865266	0.001929724
HexCer(d18:1/16:0)	8.67274E-05	5.72774E-05	1.736105792	3.6502E-08
**Downregulated**				
MG(18:1)	6.68287E-05	0.000121611	1.698389962	3.23597E-07
MG(22:4)	1.9499E-05	4.13336E-05	1.717901474	3.69537E-07
DG(18:1/18:1)	0.003802847	0.006430644	1.67435741	9.98164E-06
Cer(t14:1/24:4)	0.00021286	0.000306773	1.633731226	1.49885E-05
DG(18:1/11:0)	2.86785E-05	4.03112E-05	1.667830547	1.83293E-05
PC(P-20:0/18:2)	2.63191E-05	3.34275E-05	1.583959212	2.60475E-05
DG(22:6/22:2)	4.64786E-07	9.2068E-07	1.569274324	2.8894E-05
MG(18:2)	4.74745E-05	8.31168E-05	1.636213786	3.97438E-05
SM(d15:0/20:0)	7.81324E-06	9.92859E-06	1.573560159	7.48258E-05
TG(14:0/16:0/16:0)	0.0002281	0.000314764	1.528791154	0.000102201
PC(P-20:0/14:1)	5.95593E-05	6.96924E-05	1.562153134	0.000163043
PE(P-18:0/20:4)	6.44513E-06	9.08082E-06	1.614721071	0.000168646
PS(20:0/15:1)	7.17214E-06	1.68524E-05	1.610366809	0.000250386
TG(16:0/16:0/18:3)	2.74301E-05	3.3855E-05	1.531444256	0.000352698
PE(22:0/6:0)	2.98281E-06	5.11025E-06	1.674753549	0.00043549
PE(P-16:0/22:5)	1.20013E-05	2.08918E-05	1.545456353	0.00044156
PC(O-16:0/0:0)	3.8249E-07	5.66847E-07	1.495903496	0.000446668
PC(O-18:1/0:0)	5.35639E-06	9.10166E-06	1.660464722	0.00044843
TG(17:1/17:1/19:0)	0.000253649	0.00031972	1.501064831	0.000473077
DG(18:1/12:0)	0.000623169	0.001082651	1.551640966	0.000486342
DG(18:0/18:0)	4.21601E-05	5.76514E-05	1.650211743	0.000550865
DG(20:1/20:1)	5.96911E-05	6.98941E-05	1.417417102	0.00089955
TG(20:1/20:1/20:3)	5.3131E-06	8.60194E-06	1.458157757	0.000925138
TG(12:0/16:0/18:0)	0.00174576	0.002266135	1.498999123	0.000963483
PS(22:2/14:0)	0.00097586	0.001104228	1.441677973	0.00109107
DG(16:0/16:0)	0.001142656	0.001410798	1.599660866	0.001113963
TG(21:0/22:1/22:2)	6.95148E-06	1.31767E-05	1.423852611	0.001177453
MG(14:0)	3.88534E-06	7.44905E-06	1.625605223	0.001185778
TG(20:3/22:4/22:4)	1.00909E-05	2.05045E-05	1.404088604	0.001327215
PC(2:0/24:0)	1.41719E-06	2.69187E-06	1.50878231	0.001420451

*Preterm, mean value of preterm group; Term, mean value of term group; VIP, variable importance in projection*.

### Preterm Human Milk Contains Higher Level of PE and PC Than Term Human Milk

**Figure 3:**
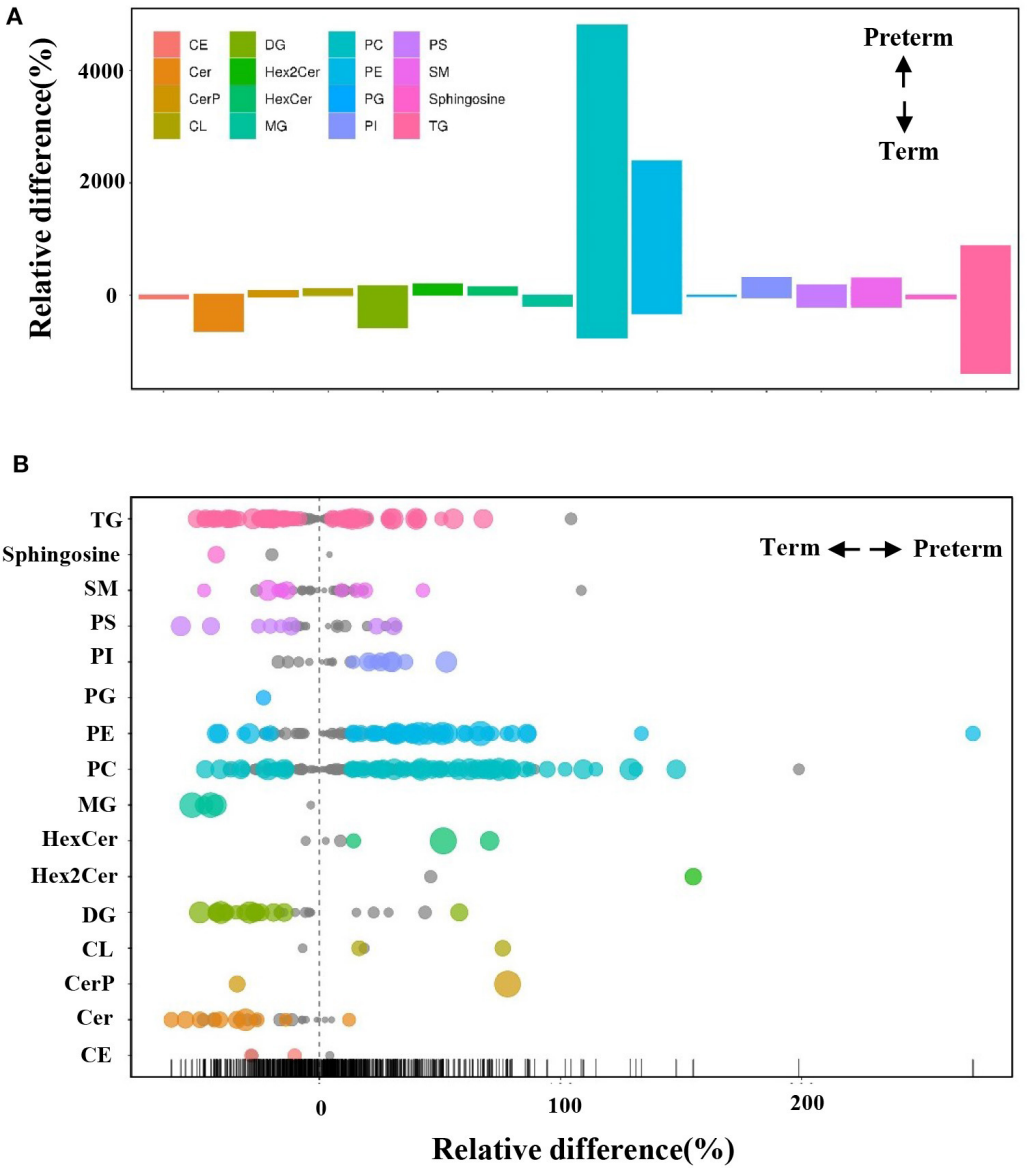
Differences in the relative percentage of significantly different lipid species between preterm and term human milk. **(A)** The bar plot shows the relative difference in the detected significantly different lipids. Each column represents a subclass of lipids. The y axis represents the relative difference of various lipids. A positive relative difference indicates that the content of this kind of substance was higher in the preterm group. A negative relative difference indicates that the content of this kind of substance was higher in the term group. The values corresponding to the top and bottom margins of the column represent the relative difference. **(B)** A bubble plot shows the relative differences in the detected lipids. Each point represents a lipid. The size of the point represents the *P*-value. Larger points represent smaller *P*-values. Colored points indicate that the *P* < 0.05.

**Figure 4:**
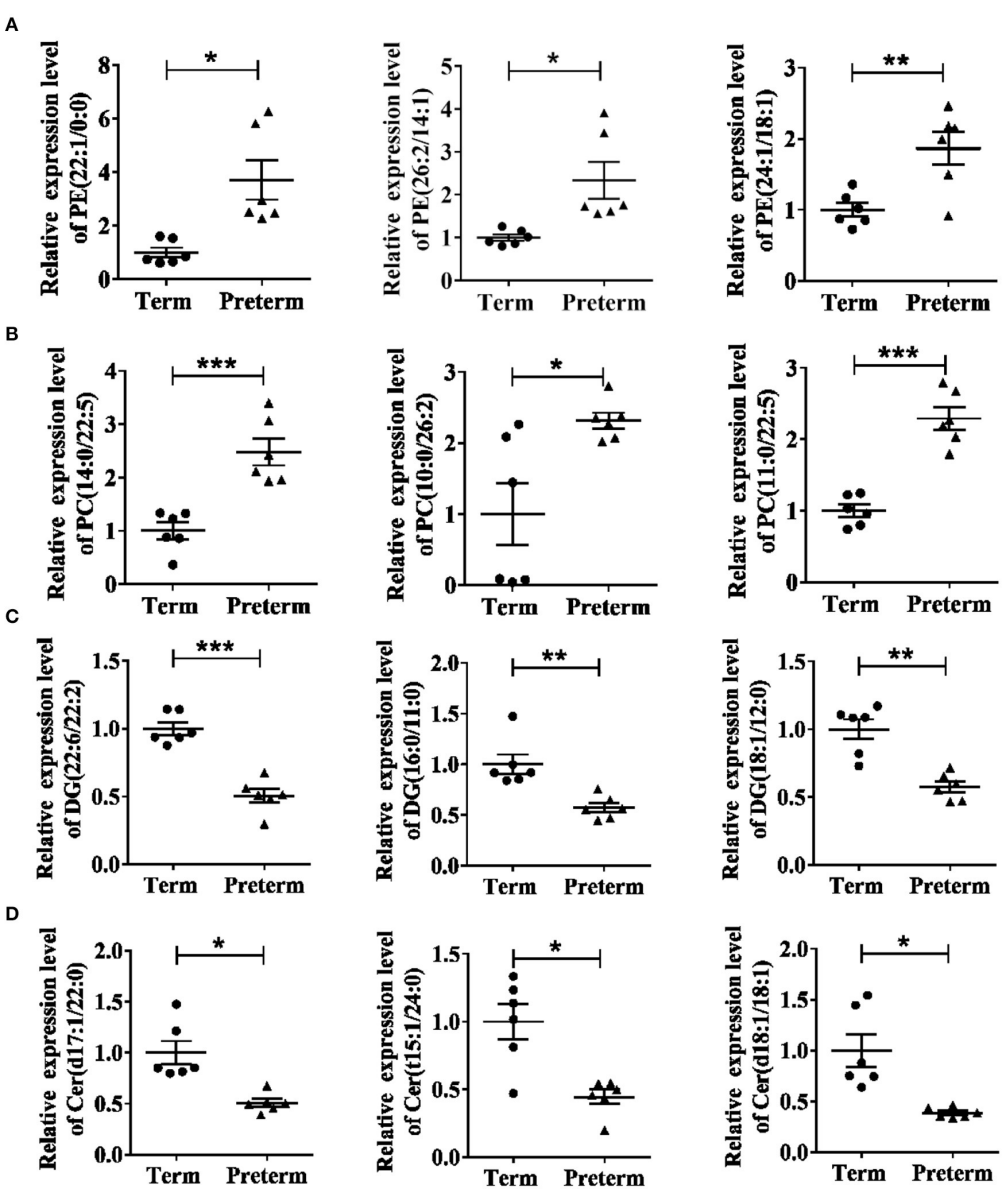
Twelve significantly different lipids in the term and preterm groups. **(A)** Top three significantly upregulated PEs in preterm group. **(B)** Top three significantly upregulated PCs in preterm group. **(C)** Top three significantly downregulated DGs in preterm group. **(D)** Top three significantly downregulated Cers in preterm group. PE, phosphatidylethanolamine; PC, phosphatidylcholine; DG, diacylglycerol; Cer, ceramide. *P < 0.05; **P < 0.01; ***P < 0.001.

The differences in the comparative proportion of identified lipid species were used to reveal the composition and distribution of lipids. The relative differences among the lipid clusters exhibited remarkable changes in the levels of lipid species in most of the inspected lipid groups. In total, 16 lipid subclasses, including triacylglycerol (TG), sphingosine, sphingomyelins (SM), phosphatidylserine (PS), phosphatidylinositol (PI), PG, PE, phosphatidylcholine (PC), monoacylglycerol (MG), DG, cardiolipin (CL), cholesteryl ester (CE), hexosylceramide (HexCer), dihexosylceramide (Hex2Cer), ceramide phosphate (CerP), and ceramide (Cer) were significantly different between the preterm and term arms (Figures 3A,B). Interestingly, PE, PC, DG, and Cer changed robustly in the two groups (Figure 3B). The top three significantly upregulated PE in the preterm arm includes PE (22:1/0:0), PE (26:2/14:1), and PE (24:1/18:1) (Figure 4A). The top three significantly upregulated PC in the preterm arm includes PC (14:0/22:5), PC (10:0/26:2), and PC (11:0/22:5) (Figure 4B). The top three significantly downregulated DG in the preterm arm includes DG (22:6/22:2), DG (16:0/11:0), and DG (18:1/12:0) (Figure 4C). The top three significantly downregulated Cer in the preterm arm includes Cer (d17:1/22:0), Cer (t15:1/24:0), and Cer (d18:1/18:1) (Figure 4D).

### The Metabolomic Pathways of the Differential Lipids Between the Term and Preterm Groups

**Figure 5:**
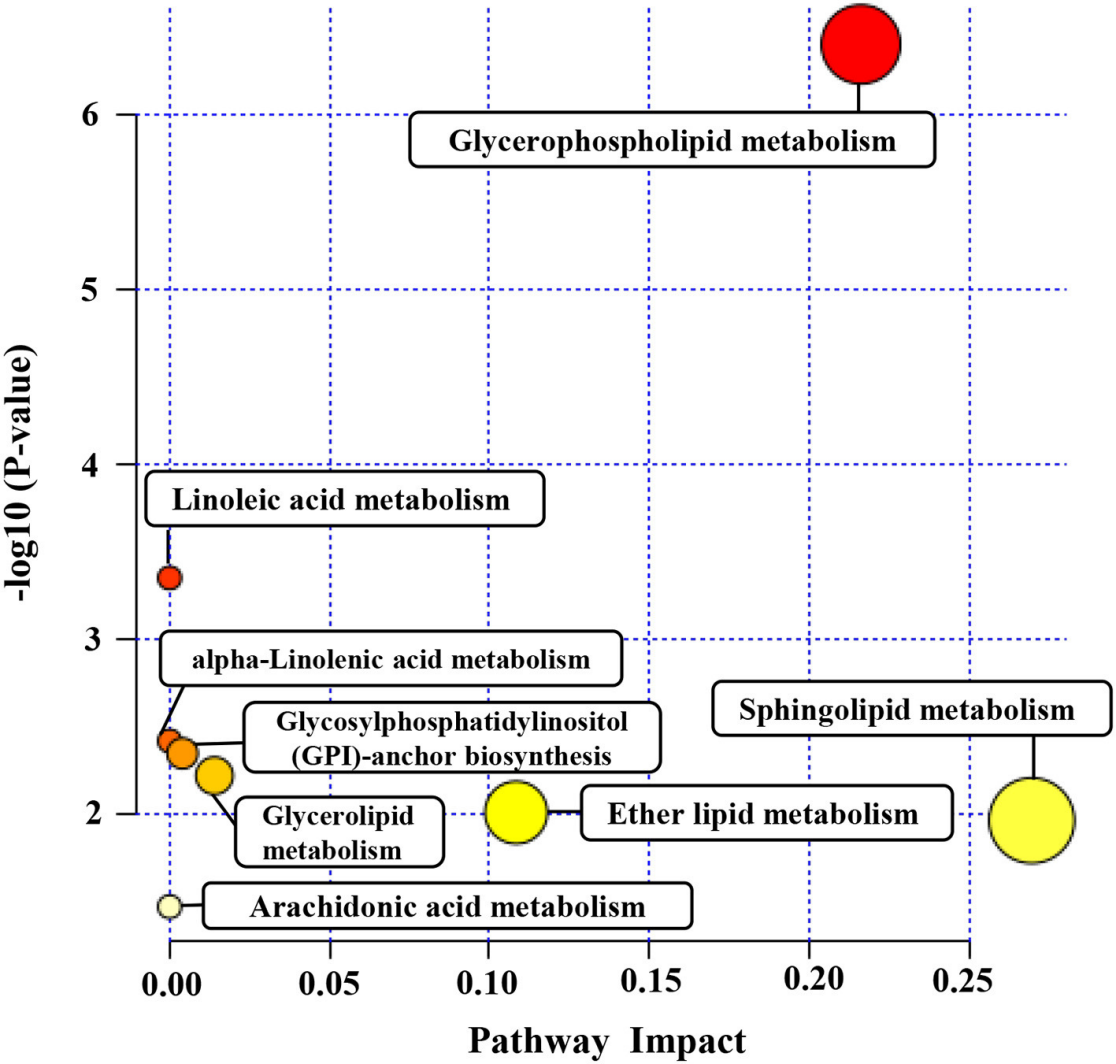
The metabolomic pathway of significantly different lipids analyzed by MetaboAnalyst. Pathway analysis of the significantly different lipids was carried out by MetaboAnalyst. The y axis represents the transformation of the original *P*-value calculated from the enrichment analysis. The x axis represents the value calculated from the pathway topology analysis.

**Figure 6:**
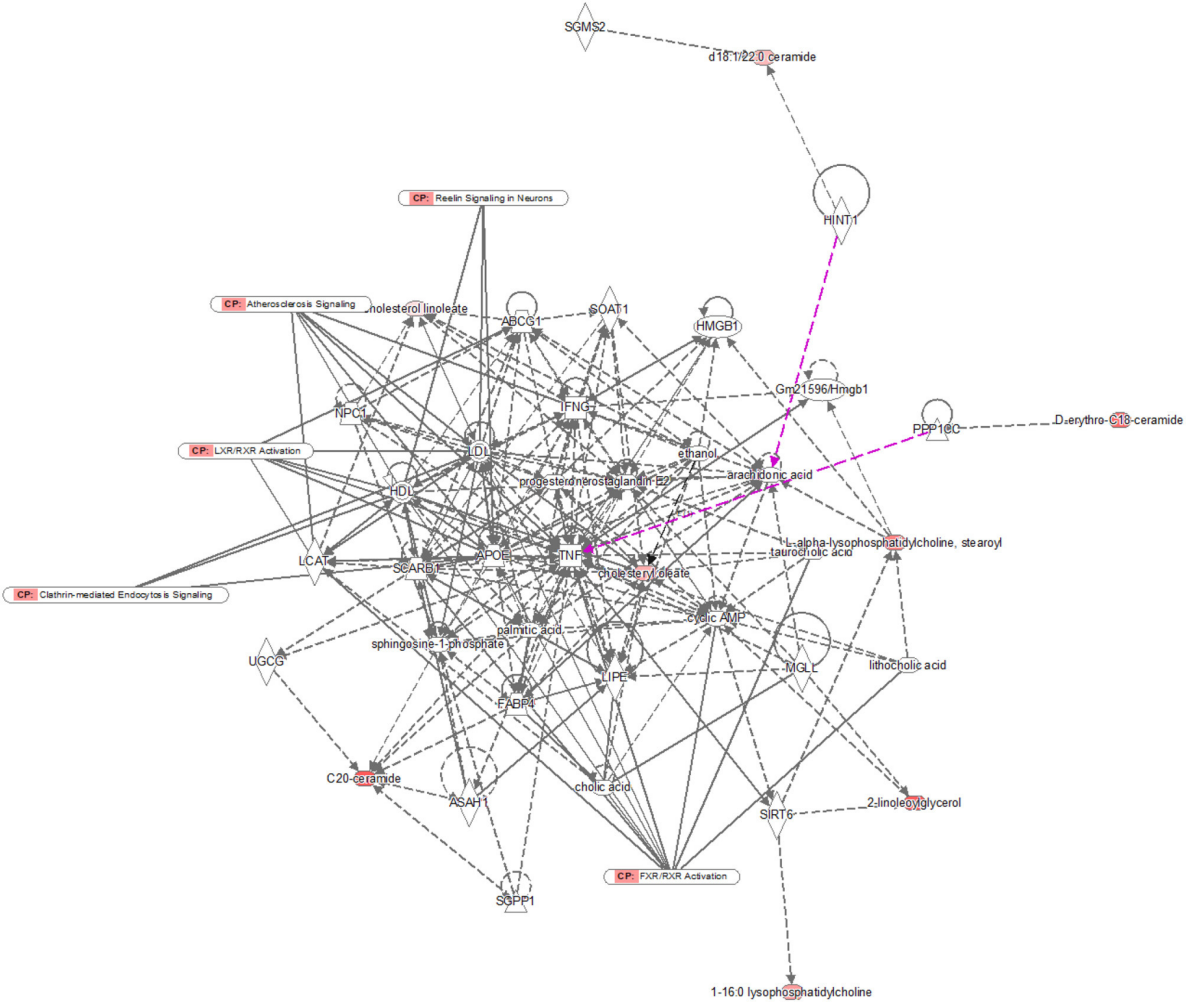
IPA prediction networks of significantly different lipids. The significantly different lipids were imported into the IPA software for pathway analysis. Direct connections are shown via solid lines, while indirect interactions are shown via dashed lines. IPA, ingenuity pathway analysis.

Pathway analysis by MetaboAnalyst was conducted to reveal the potential functions of the significantly different lipids. These lipids were predicted to participate in eight metabolic pathways, including glycerophospholipid biometabolism, glycosylphosphatidylinositol-anchor biosynthesis, glycerolipid biometabolism, ether lipid biometabolism, sphingolipid biometabolism, linoleic acid biometabolism, alpha-linolenic acid biometabolism, and arachidonic acid biometabolism (Figure 5). Additionally, the dysregulated lipids were loaded into the IPA software for biological cascade assessments. The findings of the IPA evaluation indicated that these lipids were involved in the Reelin signaling in neurons and in LXR/RXR activation (Figure 6).

## Discussion

Human milk provides essential nutrients and bioactive factors to support the organ development and immune system of newborns (2, 17). Colostrum, the fluid produced by the mammary glands over the first few postnatal days, is rich in biologically protective components; these components are present in elevated levels in the colostrum of mothers who have given birth to preterm infants than in that of mothers who have given birth to term infants (18). Additionally, mature milk becomes stable to avoid further fluctuations (19, 20). Human milk lipids are crucial sources of biological components like choline and long-chain polyunsaturated fatty acids important for neural and visual development (8). Herein, we examined the composition of lipids in the colostrum of mothers who gave birth at different gestational ages. LC-MS/MS, a powerful analytical method, was applied to identify and clarify a large number of lipids in preterm, as well as term human milk to understand the protective effects of human milk on infants.

According to the LC-MS/MS results, the constitution of colostrum lipids was found to vary at different gestational ages. Overall, 16 sub-clusters of 235 lipids were significantly different in the preterm and term human milk arms. In contrast to other traditional approaches, LC-MS/MS enabled concurrent determination and quantitation of hundreds of significantly different lipids in preterm and term human milk (8, 21). Notably, PE and PC were increased robustly in preterm human milk, while DG and Cer were decreased robustly in preterm human milk. PE, as an important constituent of mammalian cell membranes, serves a critical role in biological processes, including apoptosis, as well as cell signaling (22). Moreover, PE can also enhance memory and facilitate brain function development (23). Therefore, the high levels of remarkably different PE, including PE (22:1/0:0), PE (26:2/14:1), and PE (24:1/18:1), in preterm human milk may be strongly correlated with nervous system development in infants. PC is considered to be highly important in the development of infants, since the choline present in the neonates partly comes from PC (24). An increased choline requirement of preterm infants was reported in a previous study (25). Choline deficiency may lead to impaired neurocognitive and pulmonary development in preterm infants (25). The choline-triggered spatial memory enhancement was reported to correlate with variations in the birth, apoptosis, and migration of cells in the hippocampus during the development of the brain (26). Typically, choline translocation is introduced from the liver to other organs. In choline insufficiency, nonetheless, it is reverted toward the liver to maintain the hepatic providences, at the cost of the lungs. This may especially impact the developing lung (25). Except for being the choline source, PC facilitates the liver to regenerate from toxicity via the donation of methyl groups for hepatic recovery (27). Additionally, similar amounts of PC were added to infant formulas generated from bovine milk (28). These results suggest that PC, such as PC (14:0/22:5), PC (10:0/26:2), and PC (11:0/22:5), may serve pivotal roles in preterm infant development. DG, one of the primary lipid sub-groups in living systems and a second messenger in multiple cell activities, has been reported to hasten the β-oxidation of fatty acids, as well as influence the expression of lipid metabolism-linked genes, thereby diminishing TG levels in the serum, as well as in the liver (29, 30). The high level of DG, such as DG (22:6/22:2), DG (16:0/11:0), and DG (18:1/12:0), in term human milk may be linked to enhanced long-term health outcomes; for instance, reduced obesity, diabetes, and cardiovascular disease. Cer has many signaling roles linked to the modulation of cell growth and the initiation of apoptosis (31). In the mature, as well as the neonatal gut of term infants, mature villus cells go through apoptosis during mucosal regeneration and Cer may influence this process (32). Our data suggest that these changed subclasses of lipids may be beneficial for understanding the protective effects of preterm and term breast milk on infants.

To gain a better insight into the protective function of human milk lipids, we conducted a pathway examination to reveal the potential mechanism. Notably, the results of the pathway analysis demonstrated that the different lipids were associated with glycerophospholipid biometabolism, glycosylphosphatidylinositol-anchor biosynthesis, glycerolipid biometabolism, ether lipid biometabolism, and sphingolipid biometabolism. Glycerophospholipid metabolism and sphingolipid metabolism were identified as the most pivotal metabolic cascades with an impact value >0.2. Glycerophospholipid metabolism was reported to serve a crucial role in the regulation of monocytes and macrophages (33). The critical roles of monocytes and macrophages are the elimination of microorganisms, toxic particles, and damaged cells from inflammation sites (34). Moreover, glycerophospholipid biometabolism, a part of lipid biometabolism, participates in the PE and PC biosynthesis, which are both important for infants as described above. We speculate that glycerophospholipid metabolism may participate in the lipid dysregulation of preterm as well as term human milk. Sphingolipids are constituents of the plasma membrane and are considered regulators of cell–cell interactions, as well as cell recognition (35). Sphingolipid metabolism pathways were reported to be vital targets for therapeutics development (36, 37). A variety of studies have recently shown that affecting the enzymes involved in these sphingolipid biosynthetic pathways using inhibitors or gene deletion can attenuate the virulence of fungal pathogens; for instance, *Candida albican*s, *Cryptococcus neoformans*, and *Aspergillus spp* (38–40). Some key sphingolipid metabolites, including fatty acid 2-hydroxylase, ACER3, and ceramide synthase 2 have been proven to be critical for normal brain development and function (41–43). Notably, Reelin signaling was reported to control diverse phases of neuronal migration in the developing brain, including terminal translocation, the transition from the multipolar to bipolar neurons, and the termination of migration underneath the marginal zone (44–46). Defects in neuronal migration are thought to be involved in multiple neurological disorders, such as mental retardation (47). We speculate that Reelin signaling may participate in the improvement of neurodevelopmental outcomes for infants mediated by human milk. The integrated activation of LXR and RXR was shown to promote innate immunity to bacterial pathogens by their potential to protect macrophages from cell death caused by infection with *Escherichia coli* (48). LXR/RXR activation may provide a possible explanation for the anti-infection potential of human milk. However, these pathways need to be further validated.

In summary, the lipids in preterm and term colostrum were identified by LC-MS/MS. Significantly different lipidomic profiles and a variety of different lipids between preterm and term human milk were observed. Notably, PE and PC were increased robustly in preterm human milk, while DG and Cer were decreased robustly in preterm human milk. The significantly different lipids are involved in various pathways, including glycerophospholipid metabolism, sphingolipid metabolism, Reelin signaling in neurons, and LXR/RXR activation. These findings suggest that the presence of different lipids may explain the different effects of preterm and term human milk on infants. Our findings may provide novel insights into the mechanism of human milk lipids in neonatal development. It may offer pivotal information for the utilization of these critical constituents as nutritional, as well as functional factors in infant formula. Further studies should aim at increasing the sample size to elucidate the detailed biomechanism of these dysregulated lipids. Moreover, further studies should be conducted to validate these lipids and metabolic pathways.

## Data Availability Statement

The data that support the findings of this study are available from the corresponding author upon reasonable request.

## Ethics Statement

The studies involving human participants were reviewed and approved by Human Research Ethics Committee of Women's Hospital of Nanjing Medical University [Permission Number (2015) 88]. The patients/participants provided their written informed consent to participate in this study.

## Author Contributions

LX, WC, XW, and ZY: collected and analyzed the data. SH: designed the study and wrote the manuscript. All authors approved the final submission.

## Conflict of Interest

The authors declare that the research was conducted in the absence of any commercial or financial relationships that could be construed as a potential conflict of interest.

## Supplementary Material

Supplementary Table 1 List of the significantly different lipids.
